# Osimertinib Cost Minimization in Non‐Small Cell Lung Cancer (NSCLC) Treatment: Hypothesis Generation for a Population Pharmacokinetic Approach for Equivalent Dose Optimization of Osimertinib in Combination with Cobicistat

**DOI:** 10.1002/jcph.70085

**Published:** 2025-07-31

**Authors:** Niels Westra, Paul D. Kruithof, Sander Croes, Robin M. J. M. van Geel, Lizza E. L. Hendriks, Daan J. Touw, Jos G. W. Kosterink, Jasper Stevens, Thijs H. Oude Munnink, Paola Mian

**Affiliations:** ^1^ Department of Clinical Pharmacy and Pharmacology University Medical Center Groningen University of Groningen Groningen The Netherlands; ^2^ Department of Clinical Pharmacy and Toxicology CARIM Research Institute for Cardiovascular Diseases Maastricht University Medical Center+ Maastricht The Netherlands; ^3^ Department of Pulmonary Diseases GROW Research Institute for Oncology and Reproduction Maastricht University Medical Centre+ Maastricht The Netherlands; ^4^ Pharmaceutical Analysis Groningen Research Institute of Pharmacy University of Groningen Groningen The Netherlands; ^5^ PharmacoTherapy, Epidemiology & Economics Groningen Research Institute of Pharmacy University of Groningen Groningen The Netherlands; ^6^ Pharmacometrics Expertise Center Of the Northern Netherlands University Medical Center Groningen University of Groningen Groningen The Netherlands

**Keywords:** cobicistat, non‐small cell lung cancer, osimertinib, pharmacokinetic boosting, population pharmacokinetic modeling

## Abstract

Pharmacokinetic boosting can be a strategy to enhance osimertinib exposure and reduce treatment associated costs. The OSIBOOST trial demonstrated that it was feasible to boost low osimertinib plasma trough levels with cobicistat. The current study aims to establish the equivalent dose of cobicistat boosted osimertinib compared to osimertinib 80 mg once daily (QD) by population pharmacokinetic (popPK) modeling. A popPK model was developed on the pharmacokinetic data from the OSIBOOST study using NONMEM 7.4.4. Simulations were performed with cobicistat boosted osimertinib dosing regimens to evaluate their equivalence to the standard of osimertinib 80 mg QD. A dose level was assumed equivalent when the 90% confidence interval (CI) of the geometric mean ratios (GMR) for the area under the curve over 144 h (AUC_0‐144h_) and maximum osimertinib concentration (C_max_) were in the acceptance range of 0.8‐1.25. Cobicistat decreased osimertinib CL/F by 29.6% compared to osimertinib monotherapy (*P* < .0001). Osimertinib 80 mg 2 days on, 1 day off, boosted with cobicistat 150 mg QD was equivalent for osimertinib AUC_0‐144h_ (GMR [90% CI] = 0.96 [0.94‐0.98]) and C_max_ (GMR [90% CI] = 1.06 [1.04‐1.08]) compared to osimertinib 80 mg QD monotherapy. However, this regimen was not equivalent for AZ5104 AUC_0‐144h_ (GMR [90% CI] = 0.67 [0.66‐0.68]) and C_max_ (GMR [90% CI] = 0.74 [0.73‐0.76]). Theoretically, this reduced dose of cobicistat boosted osimertinib can potentially save approximately 33% in osimertinib treatment associated costs whilst maintaining adequate osimertinib exposure.

## Introduction

Osimertinib, a third‐generation tyrosine kinase inhibitor, is widely used as a first‐line treatment for patients with epidermal growth factor receptor (EGFR) mutated non‐small cell lung cancer (NSCLC).^1^, ^2^ Osimertinib provides substantially improved progression‐free survival and overall survival compared to first‐generation EGFR‐TKIs in metastatic NSCLC.^1^ More recently, osimertinib also demonstrated improved disease‐free survival and overall survival versus placebo in stage III disease; both in the adjuvant setting^2^, ^3^ and in the post‐chemoradiotherapy setting.^4^ However, high treatment‐associated costs of osimertinib have raised affordability concerns, particularly in low‐ and middle‐income countries, where patients have been reported to reduce osimertinib intake due to cost constraints, potentially compromising treatment efficacy.^5^, ^6^

Recently, an extensive exposure‐response model of osimertinib in NSCLC patients demonstrated that long‐term treatment with osimertinib efficacy does not improve with trough concentrations (C_min,ss_) above approximately 125 ug/L.^7^ Below this exposure threshold, data is currently too scarce to draw conclusions on osimertinib efficacy. In the same study, the authors found that the risk of left ventricular ejection fraction adverse events substantially increased with osimertinib concentration. Moreover, Agema et al.^8^ have shown that the risk of pneumonitis is increased in patients with high osimertinib trough concentrations (C_min,ss_ > 259 ug/L). Based on these findings,^7^ osimertinib exposure is potentially unnecessarily high in some patients. To lower osimertinib‐related toxicity and potentially improve costs, a current study is evaluating the efficacy and safety of osimertinib 80 mg every other day.^9^ Although this treatment strategy may potentially improve tolerability and costs, it carries a risk of undertreatment in patients with low initial osimertinib exposure.

To overcome the financial toxicity^10^ of osimertinib treatment, especially in patients with low osimertinib exposure, pharmacokinetic (PK) boosting plus dose frequency adaptation could be a promising strategy. PK boosting enhances osimertinib exposure, allowing reduced osimertinib dosing frequency to reduce treatment associated costs.^11^ Osimertinib is primarily metabolized by cytochrome P450 enzymes (CYP)3A4 and CYP3A5.^12^ Cobicistat, a relatively affordable (€109 per day in the Netherlands), selective, and strong irreversible inhibitor of the CYP3A subfamily with no intrinsic pharmacological activity, has been shown to inhibit these enzymes, thereby increasing plasma concentrations of drugs metabolized via this pathway.^13^, ^14^ PK‐boosting with cobicistat could enhance osimertinib exposure, potentially allowing for dose reductions that mitigate costs. Furthermore, the metabolism of osimertinib's active metabolite AZ5104 is also dependent on CYP3A activity, suggesting that its exposure is likely affected only to a small extent.^15^ PK‐boosting strategies using cobicistat have already been successfully employed in HIV‐treatments,^14^ and have more recently demonstrated potential in oncology,^11^ with applications in drugs such as axitinib,^16^ crizotinib,^17^ and olaparib.^18^ The OSIBOOST clinical trial has demonstrated that it was feasible to boost low plasma trough levels of osimertinib with cobicistat.^15^ The study selected and enrolled 11 patients with low osimertinib exposure (C_min,SS_ ≤ 195 µg/L), and demonstrated that cobicistat increased osimertinib exposure by 50%, without any grade ≥3 adverse events (AEs) or new safety signals.^15^

Despite the promising findings from OSIBOOST, the first part of the trial did not explore dose adjustments of osimertinib, leaving uncertainties for the moment about how best to combine PK‐boosting with reduced osimertinib dosing strategies. The current second part of the trial (i.e., with focus on osimertinib dose adaptation) is currently recruiting patients (NCT05748093 / EU CT‐number 2023‐505700‐35‐00).^19^ To help bridge this gap, the current study aims to find a^20^ cobicistat boosted osimertinib dosing regimen that provides equivalent^20^ osimertinib exposure compared to osimertinib 80 mg once daily (QD) standard of care. This will be achieved by developing a population pharmacokinetic (popPK) model to investigate the PK effect of cobicistat on osimertinib exposure. Although this model's generalizability may be limited by the small sample size and inclusion criteria in the OSIBOOST trial–particularly the C_min,SS_ ≤ 195 µg/L threshold, it may serve as a valuable exploratory step toward developing of a cost‐effective treatment strategy for osimertinib.

## Methods

### Data Collection

The data used in this study were obtained from the OSIBOOST clinical trial (NCT03858491).^15^ The trial was approved by an independent medical ethics committee (METC19‐013) and was conducted in accordance with the 1964 Helsinki declaration (and its amendments). All patients provided written informed consent. Patients were included from one academic medical center and a specialized tertiary referral center in the Netherlands (the Maastricht University Medical Centre and the Antoni van Leeuwenhoek hospital). In the OSIBOOST clinical trial, patients were selected for inclusion if they had a relatively low osimertinib trough concentration in steady state (C_min,SS_) ≤ 195 µg/L under regular oral osimertinib treatment of 80 mg QD.^15^ Steady state PK samples (pre‐dose, 0.5‐1.5 h, 2.5‐3.5 h, 7‐8 h) of osimertinib monotherapy were obtained, after which cobicistat 150 mg QD was added to the osimertinib treatment to boost its exposure. After 21 days of concomitant treatment with cobicistat (to reach steady state), PK samples (pre‐dose, 0.5‐1.5 h, 2.5‐3.5 h, 7‐8 h) of the concomitant therapy were collected. A total of 11 patients were enrolled in the OSIBOOST clinical trial, and for these patients combined, a total of 88 osimertinib and AZ5104 concentrations were determined.^15^, ^21^ Osimertinib metabolite AZ7550 was not used in this study as it was not included in the initial analysis of the OSIBOOST trial.^15^ After collection, osimertinib samples were immediately processed and analyzed by a previously validated assay.^22^, ^23^ Baseline characteristics are shown in Table 1.

**Table 1:** Baseline Characteristics of Selected Population Pharmacokinetic Models and OSIBOOST Cohort

Characteristics	(N, % or median)	OSIBOOST cohort
n	n	11
Age (years)	m	69.0
Female sex	%	63.6
Healthy subjects	%	0.0
Hist. Adenocarcinoma	%	100.0
Weight (kg)	m	78.5
Length (cm)	m	166.0
BMI (kg/m^2^)	m	23.6
BSA (m^2^)	m	1.9
Ethnicity		
Caucasian	%	100.0
Smoking status		
Never	%	36.4
Current	%	9.1
Former	%	54.5
Performance status (ECOG/WHO)		
PS: 0‐1	%	100.0
PS: 2+	%	0.0
PS: Missing	%	0.0

### PopPK Model Development

PK data were analyzed with NONMEM version 7.4.4, Perl‐speaks‐NONMEM (PsN) version 5.4.0, R version 4.4.2, and Rstudio version 2024.09.0. The packages *xpose*, *tidyvpc*, *mrgsolve*, and *ggplot2* in R were used for plotting and simulations.

A base popPK model was developed with the data from osimertinib monotherapy, using the first‐order conditional estimation method with interaction (FOCE‐I). Allometrically scaling was applied *a priori* for apparent volume of distribution (V/F), apparent osimertinib clearance (CL/F), and absorption rate constant (Ka) with power exponents of 1, 0.75, and −0.25, respectively.^24^ Parameter changes were tested with stepwise inclusion and backward elimination. A decrease of more than −3.84 ΔOFV (*P* < .05) was considered a significant improvement when a single parameter was added to nested models. For backward elimination, a stricter criterion of 6.63 ΔOFV (*P* < .01) was used when removing a parameter from the popPK model.^25^ One‐compartment and two‐compartment models were tested to best describe the data. Exponential between subject variability (BSV) on the popPK parameters was explored in a step‐wise approach. Residual error was tested as an additional, proportional, and a combined error model. The categorical covariate gender was tested on CL/F and V/F. The continuous covariate albumin was tested, after normalization on the median albumin concentration of the population, as a power model on CL/F and V/F. Osimertinib's active metabolite AZ5104 was modelled from the central osimertinib compartment to a serial metabolite compartment with a fixed metabolization rate of 25% as demonstrated previously by Brown et al.^26^ After the osimertinib base popPK model was developed, data from concomitant cobicistat treatment were further analyzed and concomitant use of cobicistat was tested as a categorical covariate proportionally on the CL/F, V/F and relative bioavailability (F).

### PopPK Model Evaluation and Validation

Developed popPK models were evaluated based on successful minimization, change in objective function value (ΔOFV), numerical diagnostics, standard goodness of fit (GOF) plots, and conditional weighted residuals (CWRES) plots. The performance of the developed popPK models was assessed with prediction‐corrected visual predictive check (pcVPC) and normalized prediction distribution error (NPDE) plots.^27^ For the final popPK model, the median parameter estimate and 95% confidence interval (CI) were obtained non‐parametric bootstrap procedure (n = 1000) and can be considered as an internal validation.^28^, ^29^ Relative standard errors (RSE%) of estimated parameters were acceptable when <30%.^30^ A condition number was considered acceptable if <20.^30^ Both η‐shrinkage and ε‐shrinkage were considered appropriate if <30%.^31^

### Simulations

Simulations (n = 1000) were performed with the final popPK model to assess the osimertinib exposure in combination with cobicistat in different dosing regimens in steady state to ascertain the individual predictions. These simulated predictions were made with the population parameters of the final popPK model. Four different dosing regimens were chosen based on expected clinical relevance. The following dosing regimens were simulated: Dose level 1; Osimertinib 80 mg QD monotherapy (reference dose), dose level 2; osimertinib 80 mg QD with cobicistat 150 mg QD, dose level 3; osimertinib 80 mg 2 days on, 1 day off, all days boosted with cobicistat 150 mg QD, and dose level 4; osimertinib 80 mg 1 day on, 1 day off, all days boosted with cobicistat 150 mg QD. Dose level 2 (osimertinib 80 mg QD with cobicistat 150 mg QD) was chosen to demonstrate the exposure‐enhancing effect of cobicistat on standard osimertinib 80 mg QD. For these four dose levels, the 5^th^, median, and 95^th^ percentile of osimertinib individual predicted concentrations were calculated and plotted. Furthermore, the area under the curve over 144 hours (AUC_0‐144h_), minimum osimertinib concentration (C_min_), and maximum osimertinib concentration (C_max_) for each dose level were estimated, their median, 5^th^ and 95^th^ percentile were calculated and their distributions were plotted.

### Equivalence of Osimertinib Exposure

To assess whether dosing regimens provided equivalent osimertinib exposure compared to osimertinib 80 mg QD, we simulated a cross‐over study.^20^ In this simulation, each virtual patient (n = 1000) sequentially received each dose level at steady‐state, after a wash out period between treatments. The geometric mean ratios (GMR) of AUC_0‐144h_ and C_max_ were calculated using osimertinib 80 mg QD monotherapy as the within patient reference. AUC_0‐144h_ was chosen as a relevant exposure parameter because the four simulated dose levels consisted of different dosing frequencies. As there is no formal definition for equivalence of exposure, we used the European Medicines Agency (EMA) bioequivalence guideline as reference, which states that 90% CI of the GMR AUC_0‐144h_ and C_max_ had to be in the acceptance range 0.8‐1.25 to ascertain equivalence.^20^ Furthermore, a provisional therapeutic window for osimertinib was used, as depicted in Figure 3a–d. This window was based on the exposure‐response analysis published by Johnson et al., where exposures (AUC) and efficacy outcomes were collected from six different phase 1 and 2 studies.^7^ Only one of these studies reported efficacy outcomes for patients with low exposure (C_min,SS_ < 100 µg/L), raising questions about the minimum effective trough concentration for osimertinib treatment. To avoid risking patients receiving potentially subtherapeutic doses, a minimum exposure (lower boundary) of C_min,SS_ 125 µg/L may be used, based on the absence of sufficient efficacy data below this threshold. For the upper boundary, Agema et al.^8^ demonstrated that patients with high exposure (i.e., C_min,SS_ > 259 µg/L) experienced increased risk of toxicity. Therefore, in this study, a provisional therapeutic window of 125‐259 µg/L was used.

### Generalizability

To evaluate broader generalizability, concomitant use of cobicistat was also incorporated as a categorical covariate proportional to the Cl/F of osimertinib and AZ5104 CL/F in the previously developed popPK model of osimertinib and AZ5104 by Brown et al. Model diagnostics and simulations were performed in the same way as described in this method section.

## Results

### PopPK Model

A serial two‐compartment PK model with first‐order absorption and elimination, and *a priori* allometric scaling on V/F, CL/F, and Ka, described the osimertinib and AZ5104 PK best. The BSV was exponentially identified on CL/F, and the residual error model was proportional. Final parameter estimates and their corresponding nonparametric 95% CI, obtained with bootstrap, from the final popPK model are shown in Table 2. Initially, Ka was fixed at a literature value of 0.24.^26^ A sensitivity analysis on the fixed value of Ka was performed, with Ka values ranging from 0.05 to 0.45, with a step size of 0.05. Increasing Ka resulted in worse ΔOFV and GOF. Decreasing the fixed Ka showed an improved ΔOFV with a decreasing V/F until the lowest Ka value, with an accompanying V/F that was lower every step. However, visual diagnostic GOF and CWRES did not improve the fit with an increasingly lower Ka. Furthermore, V/F was also estimated lower with each lower incremental step of −0.05 Ka. Because the estimates of Ka and V/F from the sensitivity analysis were near its boundary of 0, and the visual diagnostics did not improve the fit, Ka could not be identified in this data and remained fixed at the value of 0.24. BSV was identified on CL/F. Concomitant use of cobicistat was a significant covariate on bioavailability alone (*P* < .0001, ΔOFV = −59.1) but as a covariate on CL/F ΔOFV was even lower (*P* < .0001, ΔOFV = −62.6). Combining the covariate of concomitant use of cobicistat simultaneously on osimertinib CL/F and AZ5104 CL/F and/or F did not further improve the popPK model (*P* > .05). It was therefore decided to maintain the covariate on CL/F. Cobicistat boosting decreased the osimertinib CL/F by 29.6% compared to osimertinib monotherapy. The full NONMEM model code is available in Part SI, Supporting Information.

**Table 2:** Final Model Parameter Estimates and Non‐Parametric Bootstrap Results

		Bootstrap	
Parameter	Population Estimate (RSE%)	Median	95% CI
Ka (/h)^b^	0.24 (fixed)	‐	‐
V/F osimertinib (L)^b^	990 (29%)	966	669‐1859
V/F AZ5104 (L)^b^	184 (48.5%)	181	65‐7547
CL/F osimertinib (L/h)^b^	19.0 (8.5%)	19.0	16.1‐22.6
CL/F AZ5104 (L/h)^b^	47.3 (7.8%)	46.3	31.1‐55.4
Effect of cobicistat on CL/F	0.704 (3.5%)	0.694	0.639‐0.743
Proportional error	0.178 (10.6%)	0.175	0.142‐0.216
	Between subject variability (CV%)		Shrinkage (%)
BSV on CL/F osimertinib	0.0691 (21.5%)	2.5%	
Covariance^a^	0.0292		
BSV on CL/F AZ5104	0.0598 (27.2%)	2.6%	

a Correlation coefficient between the random effects of CL/F of osimertinib and AZ5104 is 0.45.

b Allometrically scaled on bodyweight of 70 kg with power exponents of 1 (V/F) 0.75 (CL/F) and −0.25 (Ka).

BSV, between‐subject variability variance; CI, confidence interval; CL/F, apparent clearance; Ka, absorption rate constant; RSE%, relative standard error; V/F, apparent volume of distribution.

### PopPK model evaluation and validation

**Figure 1:**
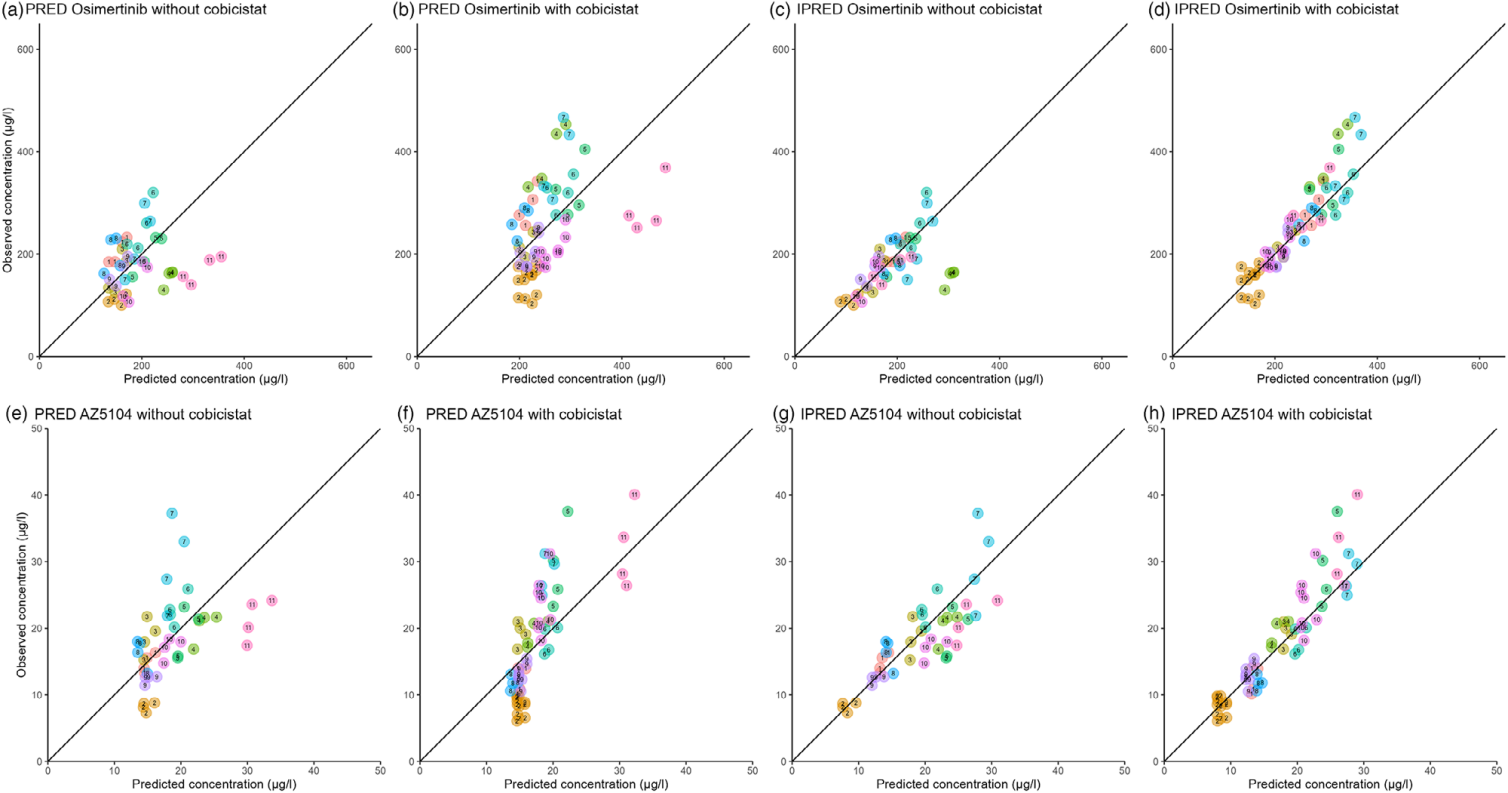
Diagnostic goodness of fit plots of osimertinib (top) and AZ5104 (bottom). Observed versus population predicted osimertinib concentrations without cobicistat (a,e); observed versus population predicted osimertinib concentrations with cobicistat (b,f); observed versus individual predicted osimertinib concentrations without cobicistat (c,g); and observed versus individual predicted osimertinib concentrations with cobicistat (d,h). The numbers in the dots represent the study ID of a participant.

Diagnostic plots indicated that the final popPK model fits the data adequately. GOF plots of the population predicted (PRED) versus observed osimertinib concentrations were scattered around the line of identity and indicated an appropriate description of the central tendency of the data (Figure 1a,b,e,f). When focusing on the PRED with concomitant cobicistat use (Figure 1b), the concentrations were distributed further away from the line of identity compared to the concentration without concomitant cobicistat use (Figure 1a). Four distinctive concentrations from patient 11 can be seen in Figure 1a,b, which appeared to be separated from the rest of the concentrations. These four concentrations all stem from one individual patient who received an increased osimertinib dose of 160 mg QD, and despite the double dose, still had a low osimertinib exposure. The individual predictions (IPRED) plotted versus observed concentrations were more closely distributed along the line of identity and showed improvement in the description of the individual trend of the data (Figure 1c,d,g,h) compared to the PRED. In the IPRED versus observed concentrations without cobicistat (Figure 1c), at higher concentrations, a slight underprediction was present. Additionally, four distinctive concentrations can be seen, which are all from patient 4, who had a relatively low bodyweight of 47 kg.

**Figure 2:**
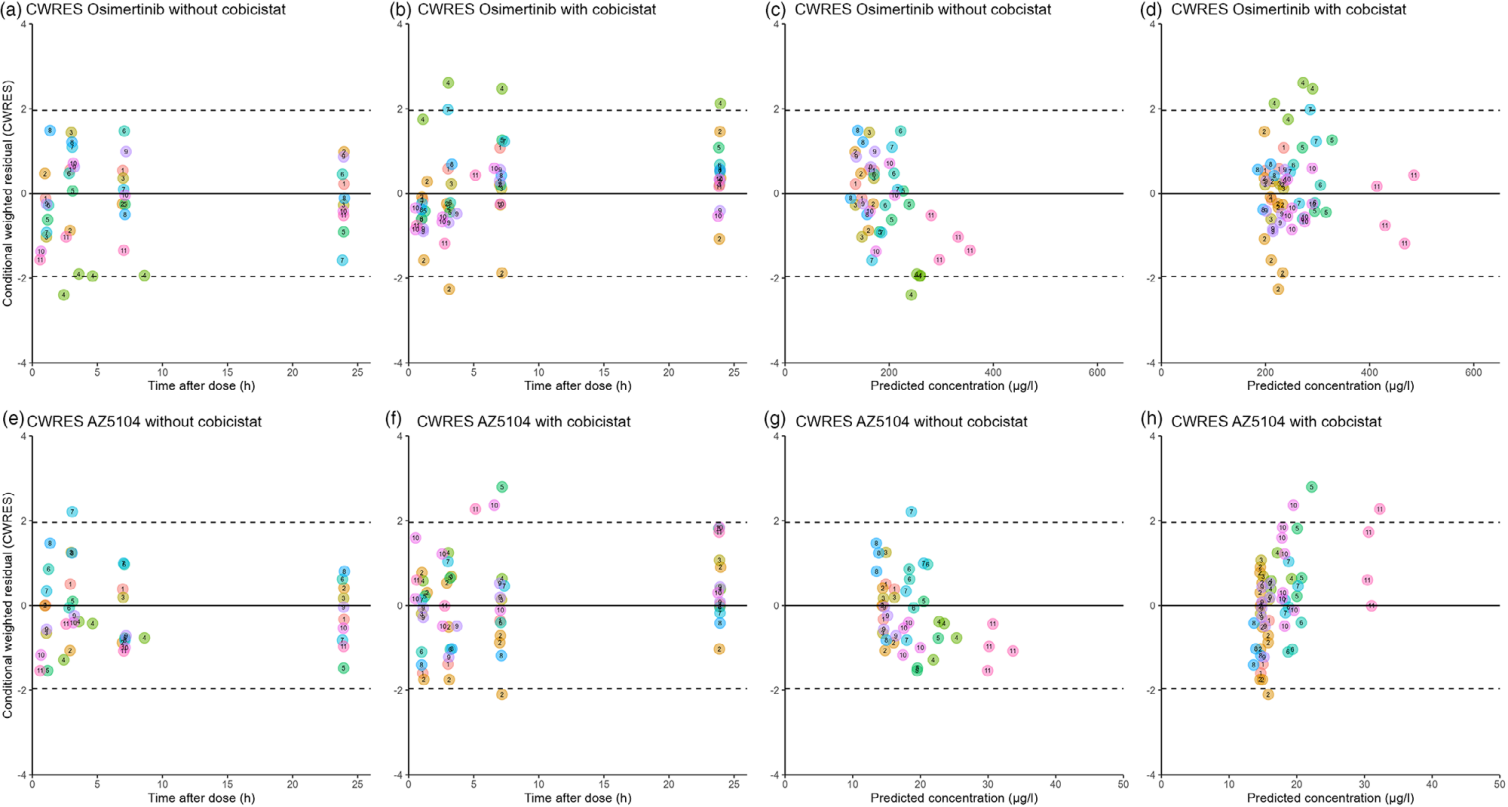
Diagnostic goodness of fit plots of osimertinib (top) and AZ5104 (bottom). CWRES versus time without cobicistat (a, e); CWRES versus time with cobicistat (b, f); CWRES versus predicted concentrations without cobicistat (c, g); and CWRES versus predicted concentrations with cobicistat (d, h). The numbers in the dots represent the study ID of a participant.

For CWRES versus time after dose of osimertinib (Figure 2a,b), the residuals before 2 h were mostly scattered below the line of identity, indicating an underprediction of early samples. CWRES versus time after dose of AZ5104 (Figure 2e,f) is evenly distributed amongst the line of identity, indicating an underprediction of early samples. CWRES of osimertinib from the final popPK model did not exhibit a trend when plotted against PRED (Figure 2c,d). However, CWRES of patients 4 and 11 are distinctively separated from the other concentrations (Figure 2c,d) and are closer to the −2 or 2 range compared to the other patients. CWRES of AZ5104 without concomitant cobicistat use were evenly distributed amongst the line of identity (Figure 2g). CWRES of AZ5104 with concomitant cobicistat use were mostly scattered above the line of identity at higher PRED, indicating overprediction at higher concentrations (Figure 2h).

Relative standard errors (RSE%) of estimated parameters ranged from 3.5% to 29% and were therefore considered acceptable. However, the RSE of AZ5104 V/F was higher at 48.5%. Coefficient of variation (CV%) of the BSV on CL/F was 21.5% and 27.5% for osimertinib and AZ5104, respectively, and therefore considered acceptable. The condition number of 2000 indicated that the popPK was overparameterized.^30^ Both η‐shrinkage and ε‐shrinkage were <10% and indicated that individual estimates reflected the true individual parameter.^31^

**Figure 3:**
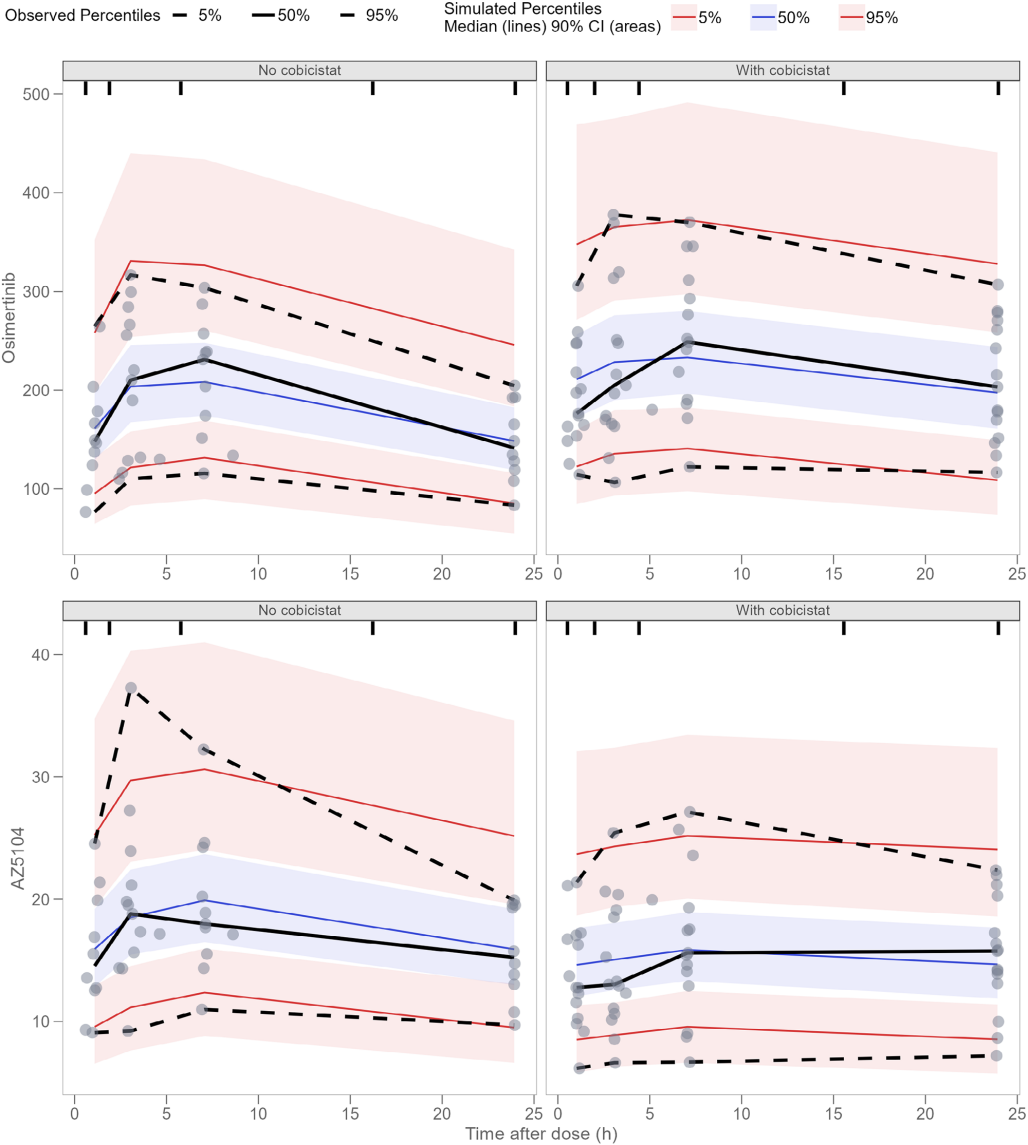
Prediction‐corrected visual predictive check (pcVPC) of osimertinib (top) and AZ5104 (bottom) versus time after dose (h) stratified on without cobicistat (left) and with cobicistat (right) treatment. The shaded areas represent the 90% confidence interval (CI) of the simulated 5th, 50th, and 95th percentile prediction‐corrected osimertinib concentrations. The black filled and dotted lines represent the 5th, 50th, and 95th percentiles of the observed prediction‐corrected osimertinib concentrations. The individual dots are the observed prediction‐corrected osimertinib concentrations.

Median lines of the simulated and observed percentiles in the pcVPC plots demonstrated the ability of the popPK model to capture the central tendency and variability of the data with and without concomitant cobicistat use (Figure 3). Simulated and observed osimertinib concentrations of the pcVPC plots were higher with concomitant cobicistat use. Simulated and observed AZ5104 concentrations of the pcVPC plots were lower with concomitant cobicistat use. 90% CIs of the 95th simulated percentile were broader with cobicistat compared to no cobicistat and therefore had more variability. Because the pcVPCs were constructed with only 11 patients, the prediction‐corrected observations were all above and under the median 5th and 95th percentile of the prediction‐corrected observations, respectively. The NPDE plots demonstrated the ability of the popPK model to accurately predict osimertinib and AZ5104 concentrations with and without concomitant cobicistat use (Figure S1).

Median parameter estimates obtained by bootstrap were all in the 0.9‐1.1 range compared to the population estimates from the popPK model. Of the 1000 bootstrap runs, 1 run did not minimize, and 4 runs were near its boundary and were therefore not included in the analysis. In conclusion, bootstrap from the popPK model indicated that the model is stable and robust.

### Simulations With Four Dose Levels

**Figure 4:**
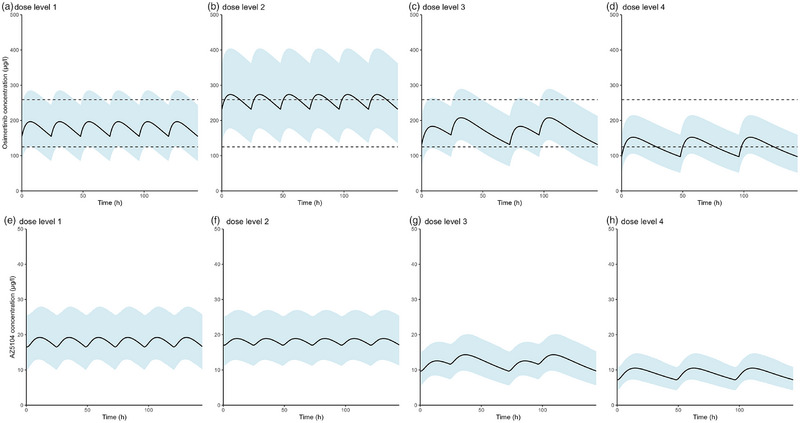
Simulated (n = 1000) osimertinib dosing regimens. The solid line represents the median osimertinib (top) or AZ5104 (bottom) concentration, the shaded area represents the 90% CI, and the dashed line is the therapeutic window of 125‐259 µg/L in the upper panel. Dose level 1 is simulated with osimertinib 80 mg QD monotherapy (a), dose level 2 is simulated with osimertinib 80 mg QD with cobicistat 150 mg QD (b), dose level 3 is simulated with osimertinib 80 mg 2 days on 1 day off, with cobicistat 150 mg QD (c), dose level 4 is simulated with osimertinib 80 mg 1 day on 1 day off, with cobicistat 150 mg QD (d).

**Figure 5:**
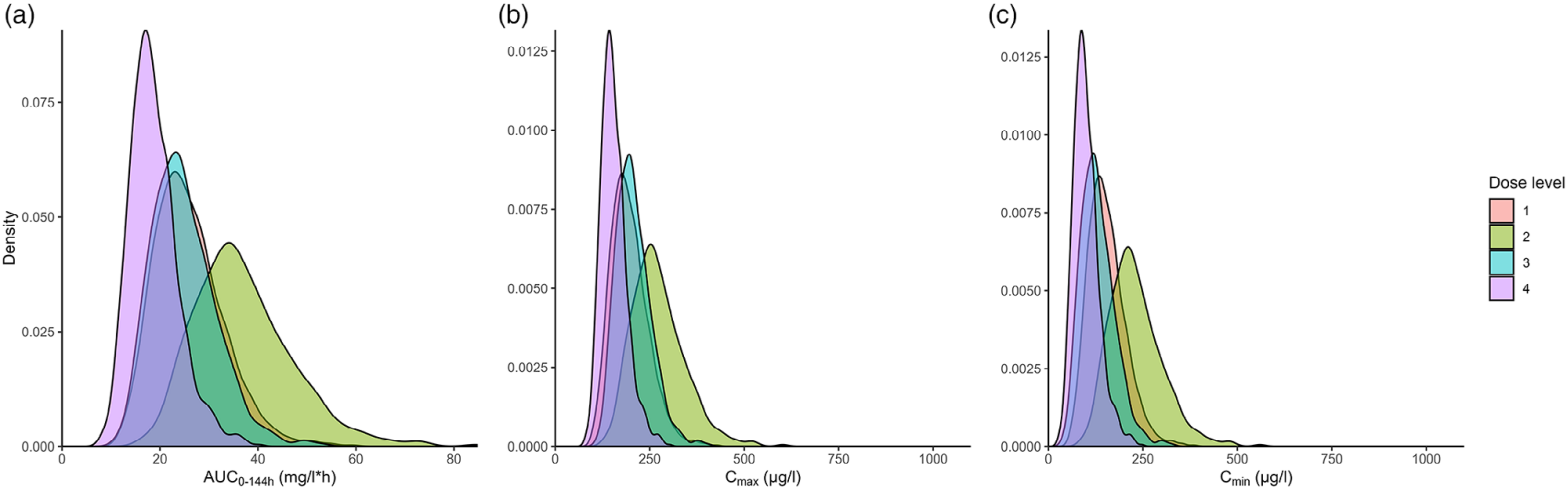
Simulated (n = 1000) distribution of osimertinib of the area under the curve 0‐144 h (a), trough plasma concentration (b), and maximum plasma concentration (c) for the four dose levels. Dose level 1 is simulated with osimertinib 80 mg QD monotherapy, dose level 2 is simulated with osimertinib 80 mg QD with cobicistat 150 mg QD, dose level 3 is simulated with osimertinib 80 mg 2 days on 1 day off, with cobicistat 150 mg QD, dose level 4 is simulated with osimertinib 80 mg 1 day on 1 day off, with cobicistat 150 mg QD.

Simulated osimertinib 80 mg dosing regimens with or without cobicistat are shown, in relation to a provisional therapeutic window of C_min,SS_: 125‐259 µg/L, in Figure 4a‐h. The associated simulated exposures are listed in Table 3. In Figure 4b, cobicistat increased the exposure of osimertinib extensively (median osimertinib AUC_0‐144h_ increased by 43%), and the median simulated osimertinib concentration was almost entirely above the provisional toxic limit for dose level 2, which would warrant an osimertinib dose alteration from a clinical perspective. In Figure 4c, dose level 3, consisting of osimertinib 80 mg 2 days on, 1 day off, boosted with cobicistat 150 mg QD resulted in equivalent exposure compared to osimertinib 80 mg QD monotherapy (median osimertinib AUC_0‐144h_ decreased by 4%). Last, in Figure 3d, a dosing regimen of osimertinib 80 mg 1 day on, 1 day off, boosted with cobicistat 150 mg QD resulted in a lower exposure compared to osimertinib 80 mg QD monotherapy (median AUC_0‐144h_ decreased by 27%). AZ5104 concentration‐time profile was not altered by concomitant cobicistat use, but was altered by the lowered dose frequency (Figure 4e–h). Simulated (n = 1000) distributions of osimertinib AUC_0‐144h_ (Figure 5a) for dose level 1 and 3 nearly overlap each other. For the distributions of C_max_ (Figure 5b), and C_min_ (Figure 5c) there seemed to be a shift where dose level 3 had a higher C_max_ and a lower C_min_. The distributions for dose levels 2 and 4 of AUC_0‐144h_ (Figure 5a), C_max_ (Figure 5b), and C_min_ (Figure 5c) were distinguishably different compared to dose level 1.

**Table 3:** Simulated (n = 1000) Osimertinib Pharmacokinetic Parameters for the Four Simulated Dose Regimens

	Osimertinib 80 mg QD Monotherapy (DL 1)	Osimertinib 80 mg QD with Cobicistat 150 mg QD (DL 2)	Osimertinib 80 mg 2 Days on, 1 Day Off with Cobicistat 150 mg QD (DL 3)	Osimertinib 80 mg 1 Day on, 1 Day Off with Cobicistat 150 mg QD (DL 4)
	Osimertinib			
Median AUC_0‐144h_ [90% CI] (mg/L × h)	25.0 [16.1‐38.6]	35.6 [23.2‐55.0]	24.0 [15.7‐36.6]	18.1 [11.9‐28.0]
Median C_max_ [90% CI] (µg/L)	190.1 [128.2‐284.2]	263.3 [177.4‐397.9]	201.2 [143.1‐288.4]	150.3 [107.5‐218.5]
Median C_min_ [90% CI] (µg/L)	148.4 [86.8‐242.2]	221.3 [135.7‐355.8]	124.9 [68.5‐211.3]	95.0 [53.1‐162.7]
GMR AUC_0‐144h_ [90% CI]	1	1.43 [1.40‐1.46]	0.96 [0.94‐0.98]	0.73 [0.71‐0.74]
GMR C_max_ [90% CI]	1	1.39 [1.37‐1.42]	1.06 [1.04‐1.08]	0.79 [0.78‐0.81]
	AZ5104			
Median AUC_0‐144h_ [90% CI] (mg/L × h)	2.6 [1.8‐3.7]	2.5 [1.7‐3.8]	1.7 [1.1‐2.5]	1.3 [0.9‐1.9]
Median C_max_ [90% CI] (µg/L)	18.4 [12.6‐27.2]	18.5 [12.3‐26.9]	13.9 [9.6‐19.9]	10.4 [7.0‐14.7]
Median C_min_ [90% CI] (µg/L)	16.2 [10.8‐24.4]	16.5 [10.8‐25.3]	9.2 [5.5‐14.8]	7.0 [4.2‐10.9]
GMR AUC_0‐144h_ [90% CI]	1	1.0 [0.99‐1.03]	0.67 [0.66‐0.68]	0.51 [0.50‐0.52]
GMR C_max_ [90% CI]	1	0.9 [0.97‐1.01]	0.74 [0.73‐0.76]	0.56 [0.55‐0.57]

AUC_0‐144h_, area under the curve over 144 h; CI, confidence interval; C_max_, maximum concentration; C_min_, minimum concentration; DL, dose level; GMR, geometric mean ratio; QD, once daily.

### Equivalence of the four simulated dose levels

Table 3 shows the simulated median AUC_0‐144h_, C_max_, C_min_, GMR AUC_0‐144h_, and GMR C_max_, for the four simulated dose levels for osimertinib and AZ5104. 90% CI of GMR of AUC_0‐144h_ and C_max_ for dose levels 2 and 4 were outside the acceptance range of 0.8‐1.25, meaning that dose level 2 and 4 are not equivalent compared to dose level 1. 90% CI GMR of osimertinib for AUC_0‐144h_ and C_max_ for dose level 3 were inside the acceptance range 0.8‐1.25, meaning that dose level 3 (osimertinib 80 mg 2 days on, 1 day off, boosted with cobicistat 150 mg QD) was equivalent to dose level 1 (osimertinib 80 mg QD monotherapy). However, the 90% CI of the GMR for AZ5104 AUC_0‐144h_ and C_max_ at dose level 3 were below the predefined acceptance range of 0.8‐1.25, indicating that dose level 3 resulted in lower AZ5104 exposure compared to dose level 1.

### Generalizability

Concomitant use of cobicistat was a significant covariate on osimertinib CL/F (*P* < .0001, ΔOFV = −63.4). Including cobicistat use as a covariate on both osimertinib and AZ5104 CL/F did not further improve the popPK model (*P* > .05). Model diagnostics and simulations of the popPK model by Brown et al. incorporating concomitant cobicistat use as a covariate on osimertinib CL/F are shown in supplementary Figures S3‐S7 and Table S1. In this model, cobicistat boosting decreased the osimertinib CL/F by 32.2% compared to osimertinib monotherapy, closely aligning with the 29.6% reduction found in our popPK model. The full NONMEM model code with its final estimates is available in Part SII, Supporting Information.

## Discussion

In this work, we explored PK‐boosting and alternative osimertinib dosing strategies as an exploratory study for designing an osimertinib treatment strategy with potentially lower costs. Based on simulations obtained from the popPK model of osimertinib with cobicistat, a dosing regimen of osimertinib 80 mg 2 days on, 1 day off, boosted with cobicistat 150 mg QD yielded an equivalent exposure compared to osimertinib 80 mg QD monotherapy. In theory, this reduced dose of osimertinib boosted with cobicistat 150 mg QD can drastically save osimertinib associated healthcare costs globally whilst maintaining adequate osimertinib exposure.

The popPK model was able to adequately describe osimertinib concentrations with and without concomitant cobicistat use. The popPK model structure and its estimates were comparable to four previously published osimertinib popPK models.^8^, ^21^, ^26^, ^32^, ^33^ Because Ka could not be estimated from our relatively small population and sparsely sampled data, its value was fixed at a literature value of 0.24.^26^ Brown et al.,^26^ Ishikawa et al.,^32^ and Agema et al.^8^ found an absorption rate constant of 0.24, 0.334, and 0.332, respectively. Using the fixed Ka literature value of 0.24 from Brown et al. seemed to be most appropriate because that study took the most samples in the absorption phase of osimertinib compared to the other three popPK models.^8^, ^26^, ^32^, ^33^ The decrease of 29.6% of osimertinib CL/F resulted in a median AUC_0‐144h_ increase of 43% when the osimertinib dose was unchanged and was in line with the results from the noncompartmental analysis, where mean AUC_0‐24h_ increased by 50% in the initial analysis of the OSIBOOST trial.^15^ Conversely, the manufacturer found a 24% increase in osimertinib exposure when combined with itraconazole and concluded that itraconazole does not have a clinically relevant drug–drug interaction (DDI).^34^ This discrepancy can be explained by the fact that itraconazole is not the optimal index inhibitor for CYP3A4 substrates; ritonavir and cobicistat have a more pronounced inhibitory effect and are considered more appropriate index inhibitors in this context.^35^

Osimertinib 80 mg 2 days on, 1 day off, boosted with cobicistat 150 mg QD (dose level 3) achieved a similar exposure when compared to osimertinib 80 mg QD monotherapy (dose level 1). Although differences in the distributions of AUC_0‐144h_, C_max_ and C_min_ for dose levels 1 and 3 were seen (Figure 5a‐c), the majority of the distributions overlap each other. This indicates that on a population basis, dose level 1 and dose level 3 were comparable. Johnson et al. used osimertinib and AZ5104 AUC as an exposure associated endpoint in their exposure‐response study; ^7^ therefore, dose level 3 should, in principle, have similar efficacy compared to dose level 1. Furthermore,^20^ osimertinib 80 mg 2 days on, 1 day off, boosted with cobicistat 150 mg QD (dose level 3) provided equivalent osimertinib exposure ^20^ compared to osimertinib 80 mg QD monotherapy (dose level 1) based on AUC_0‐144h_ and C_max_.

Reducing the osimertinib dose to 2 days on, 1 day off, boosted with cobicistat 150 mg QD can potentially reduce the osimertinib associated healthcare costs by approximately 33%. Because, cobcistat 150 mg is approximately >140 times cheaper than osimertinib 80 mg, cobicistat hardly increases costs when a boosting regimen is used. Because osimertinib 40 mg is equally expensive as 80 mg, (flat pricing^11^), a dose reduction to osimertinib 40 mg QD does not reduce costs. However, a cobicistat boosted dose reduction of osimertinib is preferably guided by TDM, which leads to additional costs. In conclusion, utilizing the relatively straightforward intervention of adding cobicisitat 150 mg QD to osimertinib 80 mg and reducing osimertinib intake to 2 days on, 1 day off (i.e., ⅔ of the standard dose) has the potential to save substantial expenses of osimertinib associated healthcare costs.

In this study, we assumed a minimum effective osimertinib exposure of C_min, ss_ = 125 ng/mL. This lower boundary was primarily based on the lack of evidence supporting efficacy at lower exposures. However, as no exposure‐efficacy relationship was found within the studied range (Cmin, ss > 125 ng/mL),^7^, ^26^ lower exposures might provide non‐inferior treatment outcomes as well. Consequently, many patients are potentially receiving higher‐than‐needed osimertinib exposures, which could lead to more treatment‐related toxicity and associated costs. In light of the FDA's Project Optimus, multiple trials are currently ongoing or in preparation with osimertinib 80 mg every other day, without boosting. Our model may require re‐evaluation when these results become available. Although lower posologies may offer more tolerable and cost‐effective treatment strategies, it should be noted that interindividual variability in osimertinib exposure is high. Therefore, lower posologies may not yield a one‐dose‐fits‐all treatment with optimal efficacy for all patients, as is currently assumed with standard osimertinib 80 mg QD.

Besides the healthcare cost‐saving potential, this popPK model can potentially also be used to help inform osimertinib treatment when co‐administration with a very strong CYP3A4 inhibitor (i.e., ketoconazol or ritonavir) is unavoidable. The model may support therapeutic drug monitoring (TDM) and predict the effect of dose frequency reductions.

This study has several limitations. First, osimertinib does not have a clear therapeutic window, so a literature‐based provisional window of 125‐259 µg/L was used in the simulation plots (Figure 4a‐d). Moreover, equivalence of the osimertinib dosing regimens was derived from the EMA guideline for bioequivalence, which is not intended to be used for boosting studies when the dosing frequency differs compared to the reference.^20^ A modified dosing frequency clearly results in an altered concentration‐time profile, making the effects of the boosted regimen with an altered frequency not straightforward to assess. However, the EMA guideline for bioequivalence can still be used as guidance because there is no formal guideline for boosting studies. Furthermore, one of the selection criteria of the used OSIBOOST dataset was that patients had a relatively low osimertinib exposure of C_min,SS_ ≤ 195 µg/L. Therefore, all patients used for the development of the popPK model had a limited osimertinib exposure, despite receiving a standard osimertinib dose (80 mg QD), and one patient even received an increased osimertinib dose (160 mg QD). This selection of patients with a low osimertinib exposure may have introduced a certain selection bias. Furthermore, high CYP3A4/A5 activity can be a reason for relatively low osimertinib exposure (our development cohort), therefore, patients with relatively low osimertinib exposure can have a more pronounced effect of cobicistat boosting. Conversely, patients with normal or low CYP3A4/CYP3A5 activity and have normal or high osimertinib exposure (more “general” cohort), can react less pronounced to boosting with cobicistat. In conclusion, the popPK model performed adequately in our dataset, however, the question remains if this popPK model can be extrapolated to a more “general” osimertinib population and has to be externally validated.

It must be noted that boosting osimertinib exposure with cobicistat can introduce some challenges and risks. An altered dosing regimen of osimertinib 80 mg 2 days on, 1 day off, boosted with cobicistat 150 mg QD is not straightforward to follow and can introduce the risk of non‐adherence, which may lead to under‐ or overexposure. Patients with relatively high osimertinib exposure can possibly have a dose reduction without the need for boosting. Boosting patients with a relatively high osimertinib exposure can possibly increase the risk of toxicity. Conversely, unnoticed decreased osimertinib exposure can lead to decreased efficacy. To mitigate the risks of toxicity or under exposure, TDM is preferably used to guide individualized osimertinib boosted. Simulations indicate that an additional osimertinib dose, skipping a day, can be introduced for patients with high initial osimertinib exposure. Ideally, the proposed 2 days on, 1 day off, boosted with cobicistat 150 mg QD dosing regimen should be prospectively tested in a clinical trial. Currently, an ongoing clinical trial is determining the optimal individualized osimertinib dose where cobicistat boosted osimertinib is one of the dose levels of that trial (OSIBOOST 2; NCT05748093).^19^ Finally, a major limitation of a boosted treatment strategy is coadministration of a very strong CYP3A4 inhibitor, such as cobicistat, introduces a risk of potentially harmful DDIs. Therefore, this treatment regimen may necessitate mitigation measures in patients using CYP3A4 or P‐glycoprotein substrate drugs. Moreover, PK boosting with cobicistat is not an option in patients using CYP3A4 or P‐glycoprotein substrate drugs for which no mitigation strategies are available. In a previous study,^18^ boosting with cobicistat was studied for olaparib treatment. The authors of this study stated that approximately 30% of patients were ineligible for inclusion because of DDIs (data not published). Therefore, co‐medication of patients considered for PK boosting using cobicistat needs to be screened for potential harmful DDIs, both upfront and during treatment. The risk of these DDIs may negatively impact the benefit/risk profile of a boosted osimertinib treatment strategy.

## Conclusions

The popPK model was able to adequately describe osimertinib and AZ5104 concentrations with and without concomitant cobicistat use. Based on simulations, a dosing regimen of osimertinib 80 mg 2 days on, 1 day off, boosted with cobicistat 150 mg QD yielded an equivalent exposure compared to osimertinib 80 mg QD monotherapy. In theory, this reduced dose of osimertinib boosted with cobicistat 150 mg QD can drastically save osimertinib associated healthcare costs globally whilst maintaining adequate osimertinib exposure.

## Author Contributions

All authors contributed to the conception and design of the research. Sander Croes and Paul D. Kruithof provided the data. Niels Westra and Paul D. Kruithof organized the database. Niels Westra performed the analyses. Paola Mian supervised the project. Niels Westra wrote the first draft of the manuscript. All authors reviewed and approved the submitted version of the manuscript.

## Conflicts of Interest

Lizza E. L. Hendriks received research funding, performed educationals/webinars, is on the advisory board, and is a local PI, all for AstraZeneca. The remaining authors have no conflicts of interest to declare.

## Funding

This study was funded by ZonMw project number 848017002.

## Supporting information



Supporting Information

## Data Availability

The NONMEM code is published in the Supporting Information. The data will be made available upon reasonable request.
