# Supplementary material for: Osimertinib Cost Minimization in Non‐Small Cell Lung Cancer (NSCLC) Treatment: Hypothesis Generation for a Population Pharmacokinetic Approach for Equivalent Dose Optimization of Osimertinib in Combination with Cobicistat
Source: J Clin Pharmacol. 2025 Jul 31;65(12):1687–98. doi: 10.1002/jcph.70085 (PMC12649290; doi:10.1002/jcph.70085)
Supplement: Supplementary file 1 — Supporting Information [file JCPH-65-1687-s001.docx]

Osimertinib Cost Minimization in non-small cell lung cancer (NSCLC) Treatment: A Population Pharmacokinetic Approach for Bioequivalent Dose Optimization of Osimertinib in Combination with Cobicistat

Niels Westra, PharmD^1^, Paul D Kruithof, PharmD^2^, Sander Croes, PharmD, PhD^2^, Robin MJM van Geel, PharmD, PhD^2^, Lizza EL Hendriks, MD, PhD^3^, Daan J Touw, PharmD, PhD^1,4^, Jos GW Kosterink, PharmD, PhD^1,5^, Jasper Stevens, PhD^1,6^, Thijs H Oude Munnink, PharmD, PhD^1^, Paola Mian, PharmD, PhD^1,6^*

AFFILIATIONS

1. Department of Clinical Pharmacy and Pharmacology, University Medical Center Groningen, University of Groningen, Groningen, The Netherlands

2. Department of Clinical Pharmacy and Toxicology, CARIM, Research Institute for Cardiovascular Diseases, Maastricht University Medical Center+, Maastricht, the Netherlands

3. Department of Pulmonary Diseases, GROW Research Institute for Oncology and Reproduction, Maastricht University Medical Centre+, Maastricht, the Netherlands

4. Pharmaceutical Analysis, Groningen Research Institute of Pharmacy, University of Groningen, Groningen, The Netherlands

5. PharmacoTherapy, Epidemiology & Economics, Groningen Research Institute of Pharmacy, University of Groningen, Groningen, The Netherlands

6. Pharmacometrics Expertise Center Of the Northern Netherlands, University Medical Center Groningen, University of Groningen, Groningen, The Netherlands

**Supplementary information**

**Part I: Additional GOF plots and model code of developed popPK model**

**
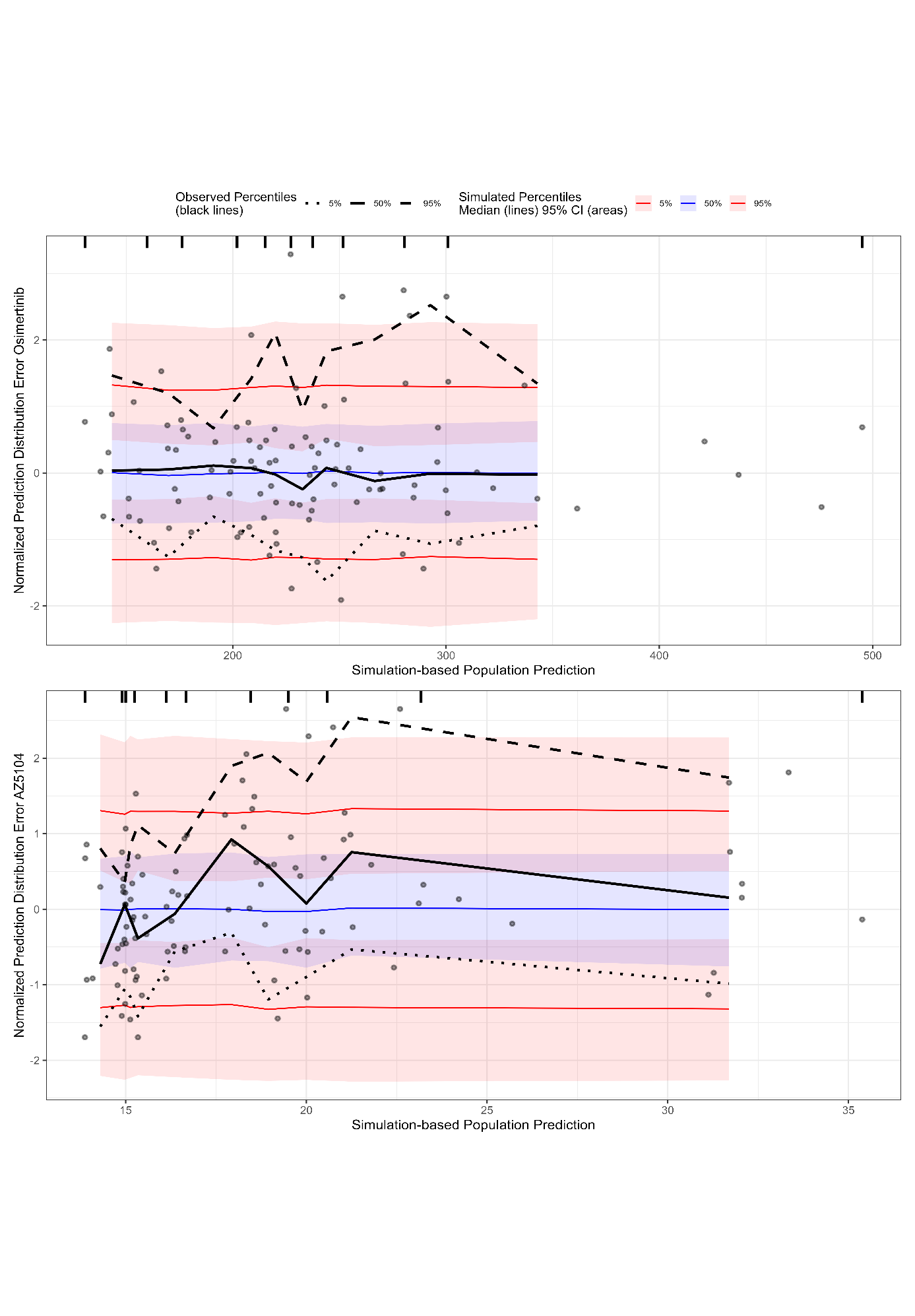
**

**Figure S1** Normalized Prediciton Distribution Error (NPDE) for Osimertinib (top) and its active metabolite AZ5104 (bottom).

**
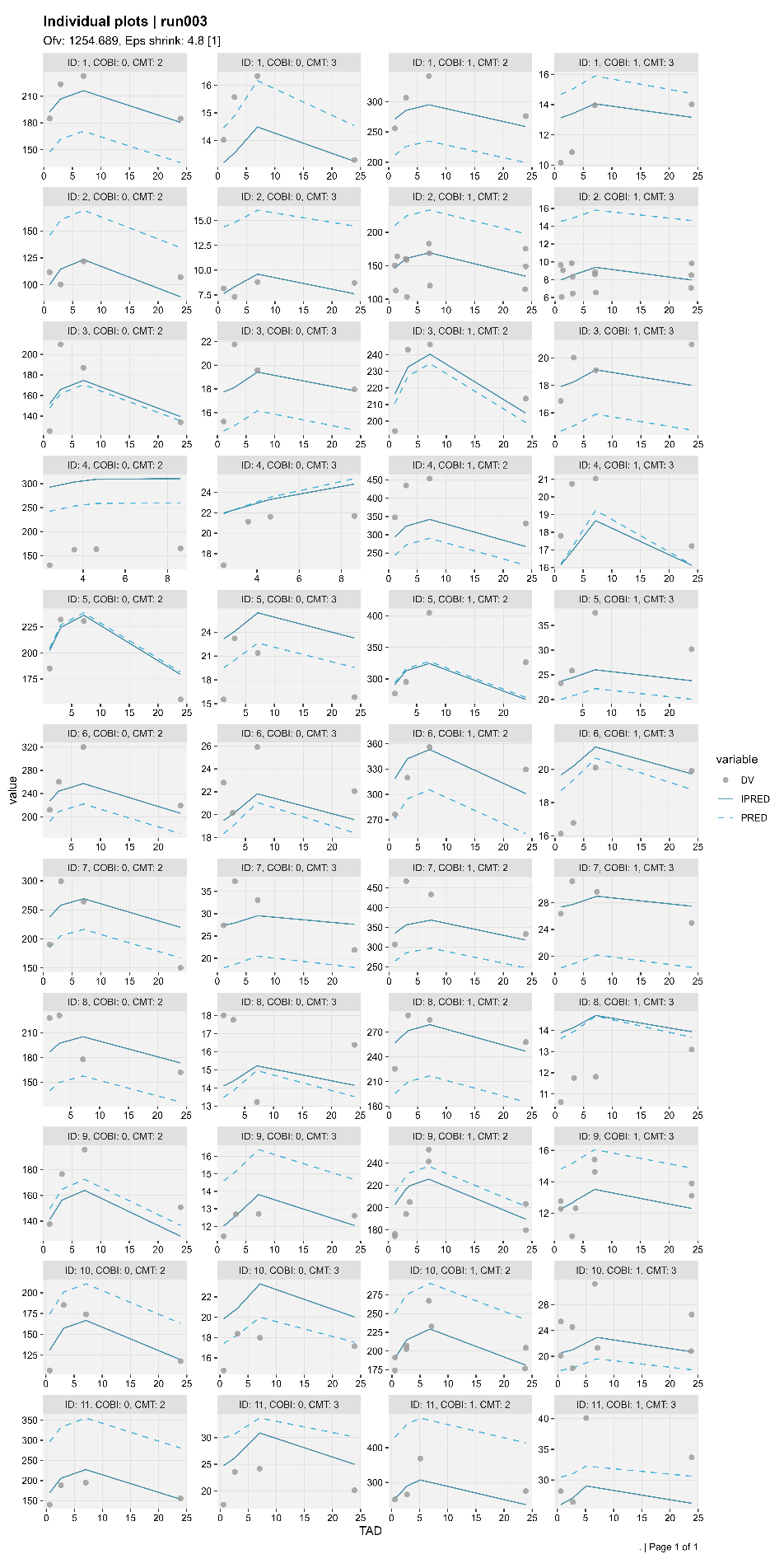
**

**Figure S2** Individual plots. COBI = 0 is no concomitant cobicistat, COBI = 1 is with concomitant cobicistat use, CMT = 2 is osimertinib, CMT = 3 is AZ5104, DV = observed concentration, IPRED = individual predicted concentration, PRED = population predicted concentration, ID is participant.

**Model code:**

; PK Osimertinib-cobicistat OSIBOOST

; Niels Westra

$PROB 1 Osimertinib-cobicistat PK model

$SUBROUTINES ADVAN6 TOL=6

$INPUT C ID INACT DAT1=DROP TIME TAD AMT ADDL II DV MDV COBI CMT EVID CRP THROMBO HEMOGLOB ALKP ALB SEX BW

$DATA OSIMV7.csv IGNORE=C IGNORE(INACT=1) ; IGNORE(INACT=1) were 7 additional t=24h TDM observations and were not sampled according to protocol and where therefore excluded from analysis.

$MODEL

NCOMP = 3

COMP (ABSORB,DEFDOSE) ;1 ABSORB

COMP ;2 PARENT

COMP ;3 METABOLITE

$PK

KA = THETA(2)*((BW/70)**(-0.25)) ;absorption constant

CL1 = THETA(3) * ((BW/70)**0.75)* (THETA(7)**COBI) * EXP(ETA(1)) ;Clearance parent (L/h)

V1 = THETA(5)*(BW/70) ;Vparent (L)

CL2 =THETA(4)*((BW/70)**0.75)* EXP(ETA(2)) ;Clearance metabolite (L/h)

V2 = THETA(6)*(BW/70) ;Vmetabolite (L)

K20 = (CL1/V1)

K30 = (CL2/V2)

K23 = K20 * 0.25

S2 = V1/1000 ;Scaling compartment

S3 = V2/1000 ;Scaling compartment

$THETA

(0, 0.178) ;1 prop error

(0.24) FIX ;2 Ka /h

(0, 19) ;3 CLparent L/h

(0, 47.3) ;4 CLmetabolite L/h

(0, 990) ;5 Vparent L

(0, 184) ;6 Vmetabolite L

(0.704) ;7 COV COBI CL

$OMEGA BLOCK(2)

0.0691 ;1 IIV CL parent

0.0292 0.0598 ;1 IIV CL metabolite

$DES

DADT(1)=-KA*A(1) ;absorption compartment

DADT(2)=(A(1)*KA) - (A(2)*K20) ;parent compartment

DADT(3)=(A(2)*K23) - (K30*A(3)) ;metabolite compartment

$SIGMA

1 FIX

$ERROR

IPRED = F

IRES = DV-IPRED

W = IPRED*THETA(1) ; IPRED*THETA(1)+THETA(2)

IF (W.EQ.0) W = 1

IWRES = IRES/W

Y= IPRED+W*ERR(1)

$EST METHOD=1 MAXEVAL=9999 SIG=3 PRINT=1 NOABORT POSTHOC INTERACTION

$COV PRINT=E

$TABLE ID TIME TAD PRED IPRED COBI RES CWRES IWRES EVID MDV CMT NOPRINT ONEHEADER FILE=sdtab006

**Part II: Results and model code of popPK model by Brown et al with the addition of concomitant cobicistat use as covariate.**

**
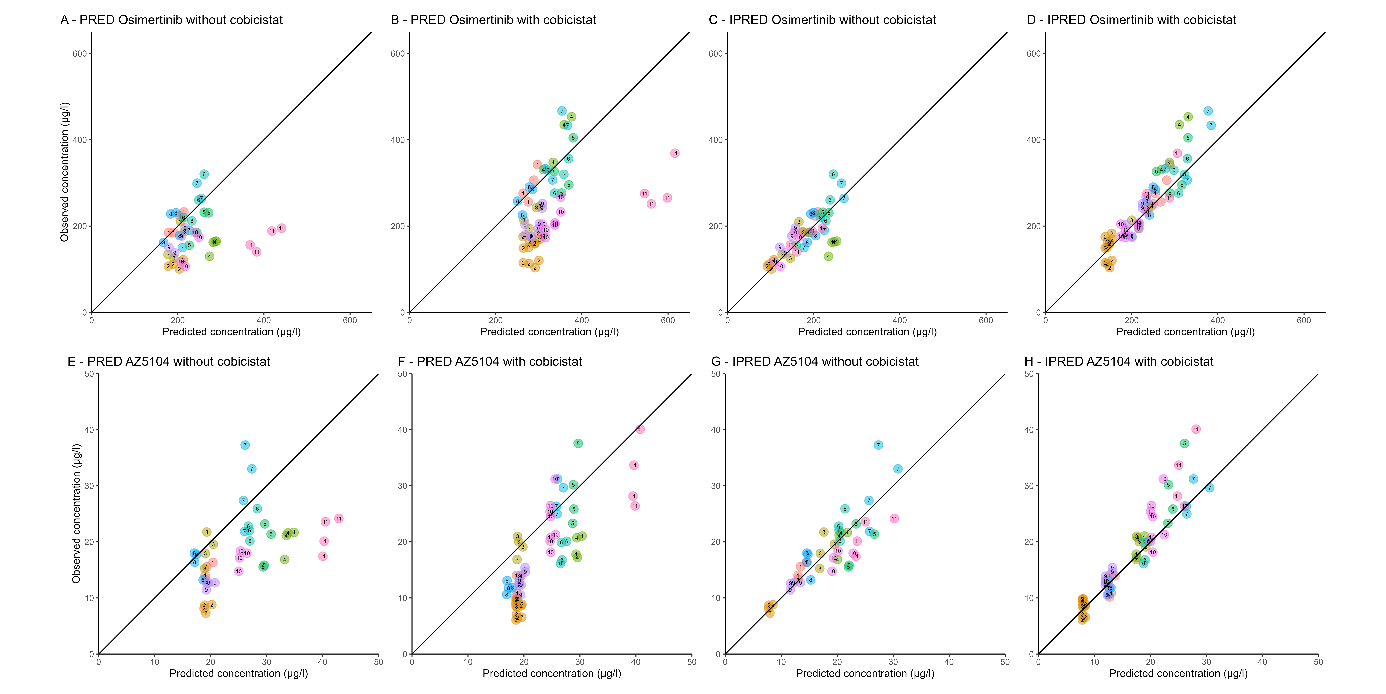
**

**Figure S3** Diagnostic goodness of fit plots for osimertinib and AZ5104. The numbers in the dots represent the study ID of a participant.


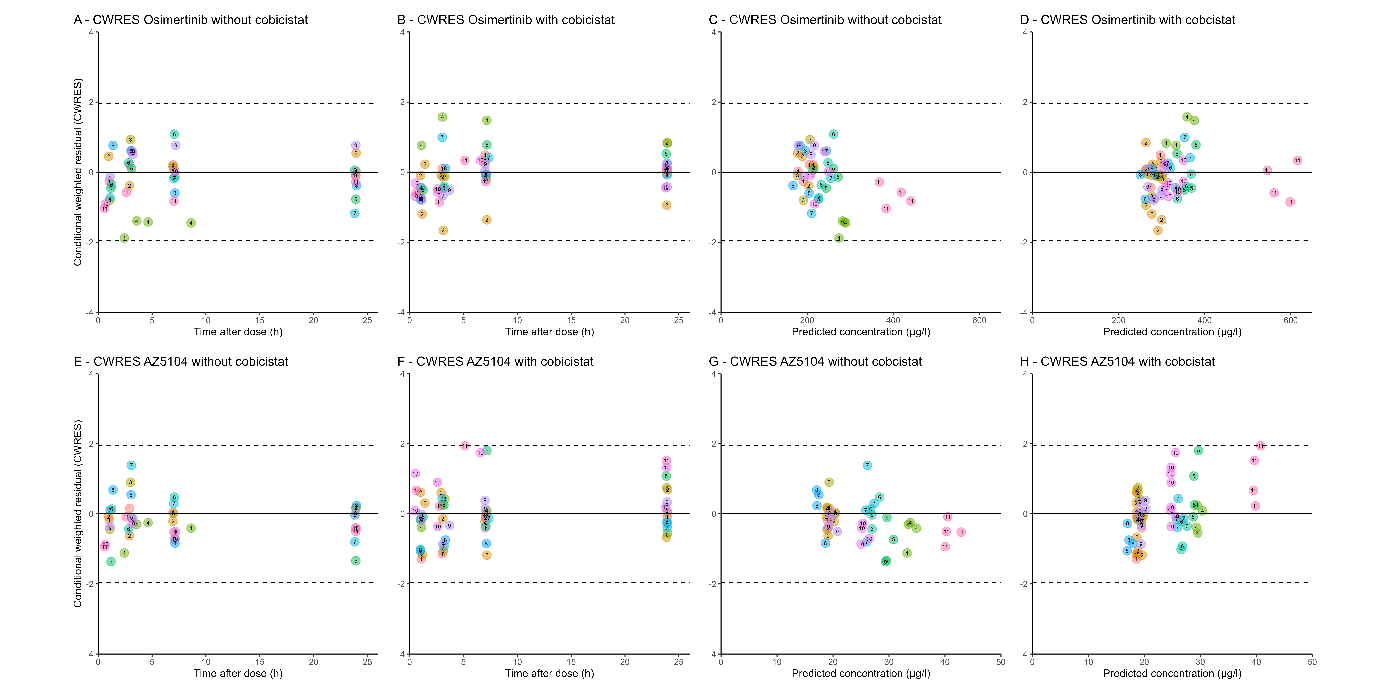


**Figure S4** Diagnostic goodness of fit residual plots for osimertinib and AZ5104. The numbers in the dots represent the study ID of a participant.


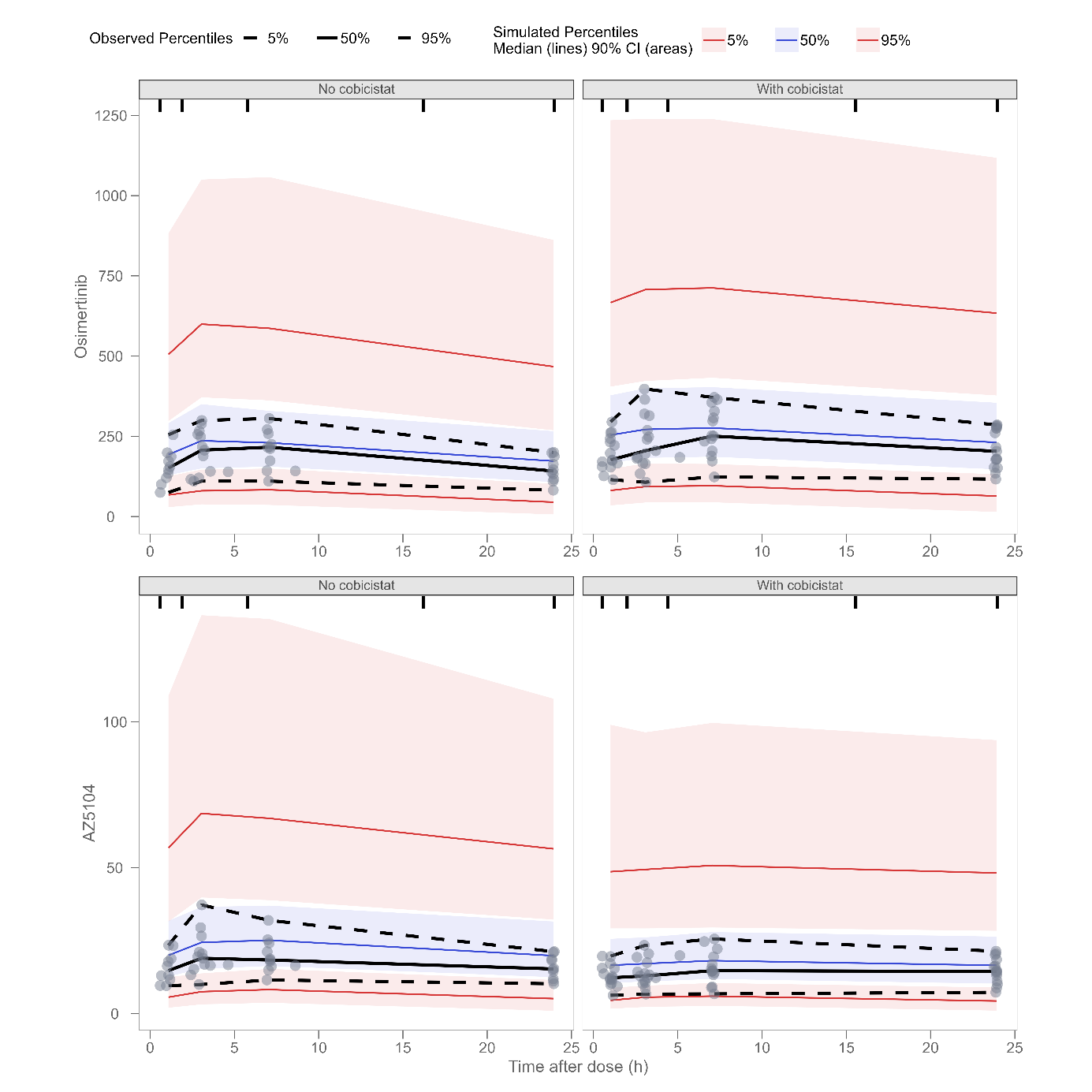


**Figure S5** Prediction-corrected visual predictive check (pcVPC) of osimertinib (top) and AZ5104 (bottom) versus time after dose (h) stratified on without cobicistat (left) and with cobicistat (right) treatment. The shaded areas represent the 90% confidence interval (CI) of the simulated 5th, 50th, and 95th percentile prediction-corrected osimertinib concentrations. The black filled and dotted lines represent the 5th, 50th, and 95th percentiles of the observed prediction-corrected osimertinib concentrations. The individual dots are the observed prediction-corrected osimertinib concentrations.


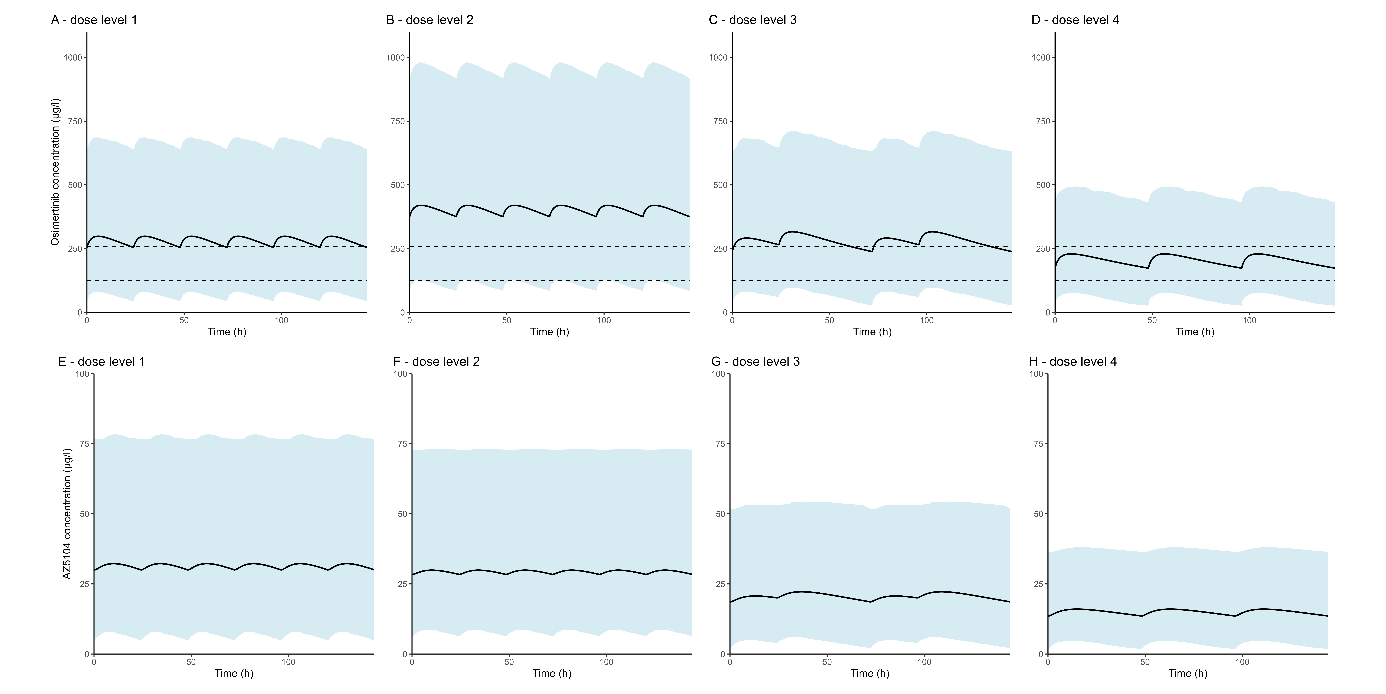


**Figure S6** Simulated (n = 1000) osimertinib dosing regimens. The solid line represents the median osimertinib or AZ5104 concentration, the shaded area represents the 90% CI, and the dashed line is the therapeutic window of 125 – 259 μg/L in the upper panel. Dose level 1 is simulated with osimertinib 80 mg QD monotherapy (A & E), dose level 2 is simulated with osimertinib 80 mg QD with cobicistat 150 mg QD (B & F), dose level 3 is simulated with osimertinib 80 mg 2 days on 1 day off, with cobicistat 150 mg QD (C & G), dose level 4 is simulated with osimertinib 80 mg 1 day on 1 day off, with cobicistat 150 mg QD (D & H).


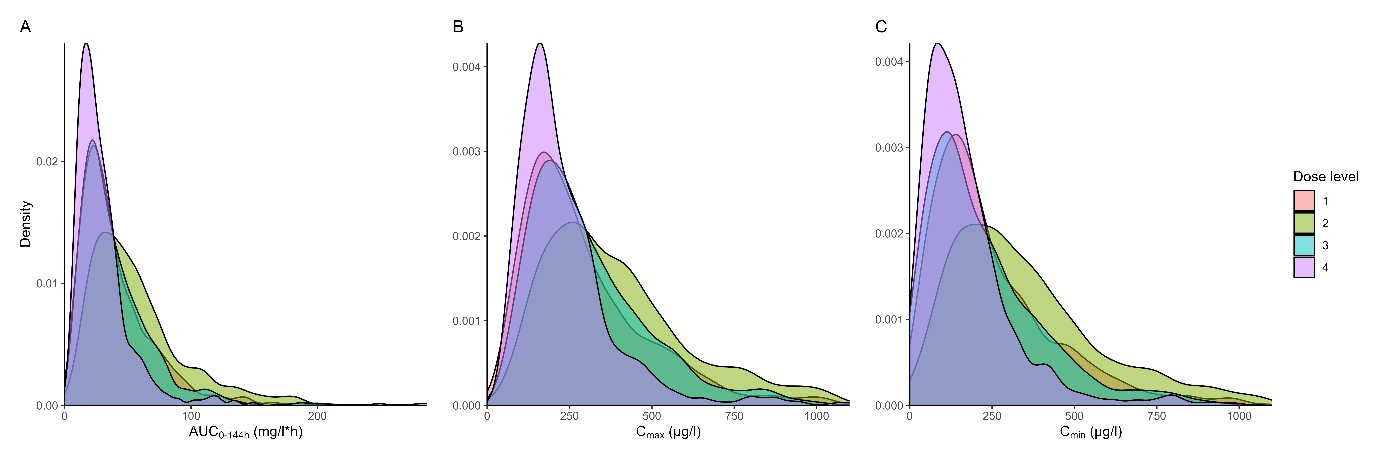


**Figure S7** Simulated (n = 1000) distribution of the area under the curve 0 - 144 h (A), trough plasma concentration (B), and maximum plasma concentration (C) for the 4 dose levels. Dose level 1 is simulated with osimertinib 80 mg QD monotherapy, dose level 2 is simulated with osimertinib 80 mg QD with cobicistat 150 mg QD, dose level 3 is simulated with osimertinib 80 mg 2 days on 1 day off, with cobicistat 150 mg QD, dose level 4 is simulated with osimertinib 80 mg 1 day on 1 day off, with cobicistat 150 mg QD.

**Table S1** Simulated (n = 1000) osimertinib pharmacokinetic parameters for the four simulated dose regimens.

|  | Osimertinib 80 mg QD monotherapy (DL 1) | Osimertinib 80 mg QD with cobicistat 150 mg QD (DL 2) | Osimertinib 80 mg 2 days on, 1 day off with cobicistat 150 mg QD (DL 3) | Osimertinib 80 mg 1 day on, 1 day off with cobicistat 150 mg QD (DL 4) |
| --- | --- | --- | --- | --- |
| Median AUC_0-144h_ [90% CI] (mg/l*h) | 32.8 [10.2-97.4] | 46.8 [15.3-142.7] | 31.0 [9.7-100.4] | 22.7 [7.9-73.8] |
| Median C_max_ [90% CI] (μg/L) | 251.5 [89.1-699.4] | 344.2 [124.3-1012] | 258.0 [97.1-742.8] | 188.2 [73.2-532.3] |
| Median C_min_ [90% CI]  (μg/L) | 202.1 [42.0-644.9] | 298.1 [81.6-982.3] | 173.7 [24.7-645.3] | 129.4 [25.4-474.3] |
| GMR AUC_0-144h_ [90% CI] | 1 | 1.45 [1.38-1.52] | 0.96 [0.91-1.01] | 0.72 [0.69-0.77] |
| GMR C_max_ [90% CI] | 1 | 1.40 [1.34-1.47] | 1.05 [1.00-1.10] | 0.78 [0.74-0.81] |

DL = dose level, AUC_0-144h_ = the area under the curve over 144 hours, C_min_ = minimum osimertinib concentration, C_max_ = maximum osimertinib concentration, GMR = geometric mean ratio, CI = confidence interval.

**Model code:**

; PK Osimertinib-cobicistat reproduced from Brown et al 2017 and added cobicistat as covariate (DOI:10.1111/bcp.13223)

; Niels Westra

$PROB 1 Osimertinib-cobicistat PK model

$SUBROUTINES ADVAN6 TOL=6

$INPUT C ID INACT DAT1=DROP TIME TAD AMT ADDL II DV MDV COBI CMT EVID CRP THROMBO HEMOGLOB ALKP ALB SEX BW

$DATA OSIMV7.csv IGNORE=C IGNORE(INACT=1); IGNORE(INACT=1) were 7 additional t=24h TDM observations and were not sampled according to protocol and where therefore excluded from analysis.

$MODEL

NCOMP = 3

COMP (ABSORB,DEFDOSE) ;1 ABSORB

COMP ;2 PARENT

COMP ;3 METABOLITE

$PK

KA = THETA(3)* EXP(ETA(3)) ;absorption constant

CL1 = (THETA(4)*((BW/62)** THETA(8)))*(THETA(12)**COBI)*EXP(ETA(1)) ;Clearance parent (L/h)

V1 = (THETA(5)* ((BW/62)** THETA(9))*((ALB/39)** THETA(11)))*EXP(ETA(4)) ;Vparent (L)

CL2 =(THETA(6)* ((BW/62)** THETA(10)))*EXP(ETA(2)) ;Clearance metabolite (L/h)

V2 = THETA(7)* EXP(ETA(5)) ;Vmetabolite (L)

K20 = (CL1/V1)

K30 = (CL2/V2)

K23 = K20 * 0.25

S2 = V1/1000 ;Scaling compartment

S3 = V2/1000 ;Scaling compartment

$THETA

(0.244) FIX ;1 prop error

(0.105) FIX ;2 Additve error

(0.24) FIX ;3 Ka /h

(14.2) FIX ;4 CLparent L/h

(986) FIX ;5 Vparent L

(31.5) FIX ;6 CLmetabolite L/h

(207) FIX ;7 Vmetabolite L

(0.56) FIX ;8 cov BW on CLp*

(0.65) FIX ;9 cov BW on V1

(0.99) FIX ;10 cov BW on CLm*

(1.33) FIX ;11 cov ALB on V1

(0.678) ;12 cov cobi on CLp

$OMEGA BLOCK(2) 0.46

0.44 0.52 ;1 IIV CL1 parent

0 0 0.89 ;2 IIV CL2 metabolite

$OMEGA

0.52 FIX ;3 IIV Ka

0.62 FIX ;4 IIV V1 parent

$DES

DADT(1)=-KA*A(1) ;absorption compartment

DADT(2)=(A(1)*KA) - (A(2)*K20) ;parent compartment

DADT(3)=(A(2)*K23) - (K30*A(3)) ;metabolite compartment

$SIGMA

1 FIX ;

$ERROR

IPRED = F

IRES = DV-IPRED

W = IPRED*THETA(1)+THETA(2)

IF (W.EQ.0) W = 1

IWRES = IRES/W

Y= IPRED+W*ERR(1)

$EST METHOD=1 MAXEVAL=9999 SIG=3 PRINT=1 NOABORT POSTHOC INTERACTION

$COV PRINT=E

$TABLE ID TIME TAD PRED IPRED RES CWRES IWRES EVID MDV CMT COBI NOPRINT ONEHEADER FILE=sdtab005
